# Federating governance, access and infrastructure to support researcher use of synthetic data

**DOI:** 10.23889/ijpds.v9i5.2603

**Published:** 2024-09-18

**Authors:** Katherine O'Sullivan, Jackie Caldwell, Christian Cole, Kathy Harrison, Charlie Mayor, Antonietta Chaliou, Jaroslaw Dymiter, Stacey Dawson, Diane Brown, Katie Wilde

**Affiliations:** 1 University of Aberdeen; 2 Public Health Scotland; 3 University of Dundee; 4 University of Edinburgh; 5 NHS Greater Glasgow & Clyde

Synthetic Data has the potential to improve efficiency of data analysis for researchers. However, there is no standard approach to synthetic data governance, access controls or infrastructure requirements, and researchers may face inconsistencies in how they can access or use synthetic data across trusted research environments. We present a federated solution taken by the Scottish Safe Haven Network to address these barriers to facilitate researcher use of synthetic data.

We documented and evaluated existing governance pathways, access controls and infrastructure design for non-synthetic data across the Network, recognising uniformity and establishing equivalence using the 5 Safes framework, ISO27001 standards and the SATRE TRE specification. We also interviewed current and potential researchers using our trusted research environments to identify common use cases for accessing synthetic data. We then mapped researcher requirements against the documented equivalencies, validating with current and prospective users.

We identified several use cases: to undertake feasibility studies, to understand dataset structure and format and to write analysis code whilst waiting on the project-specific data to be provided. By mapping the use cases onto existing governance and access processes and infrastructure designs, we were able to agree to a standard application process, access control mechanism, and infrastructure platform across the Network to provide a consistent process for researchers.

A federated approach to synthetic data access will improve the speed at which research can be conducted as well as improving the transparency and consistency of data governance and access across organisations, ultimately improving the experience for researchers using TREs.

